# Supplementary material for: Disturbance of Immune Microenvironment in Androgenetic Alopecia through Spatial Transcriptomics
Source: Int J Mol Sci. 2024 Aug 20;25(16):9031. doi: 10.3390/ijms25169031 (PMC11354591; doi:10.3390/ijms25169031)
Supplement: Supplementary file 1 [file ijms-25-09031-s001.zip › ijms-3065362-supplementary.pdf]

Supplementary Material

Disturbance of immune microenvironment in androgenetic alopecia through spatial transcriptomics

Sasin Charoensuksira [1], Supasit Tantiwong [1], Juthapa Pongklaokam [1], Sirashat Hanvivattanakul [2], Piyaporn Surinlert [2,6], *Aungkana Krajarng* [2], Wilai Thanasarnaksorn [1,4], Suradej Hongeng [5] and Saranyoo Ponnikorn [1,2,3*]

[1] Division of Dermatology, Chulabhorn International College of Medicine, Thammasat University, Pathum Thani, Thailand

[2] Chulabhorn International College of Medicine, Thammasat University, Pathum Thani, Thailand

[3] Thammasat University, Pattaya Campus, Thailand

[4] Division of Dermatology, Faculty of Medicine, Ramathibodi Hospital, Mahidol University, Bangkok, Thailand

[5] Division of Hematology and Oncology, Department of Pediatrics, Faculty of Medicine Ramathibodi Hospital, Mahidol University, Bangkok, Thailand

[6] Research Unit in Synthesis and Applications of Graphene, Thammasat University, Pathum Thani, Thailand

* Saranyoo Ponnikorn : saranyoo@tu.ac.th, ponnikorn.s@gmail.com

1 Supplementary Figures and Tables

1.1 Supplementary Figures.

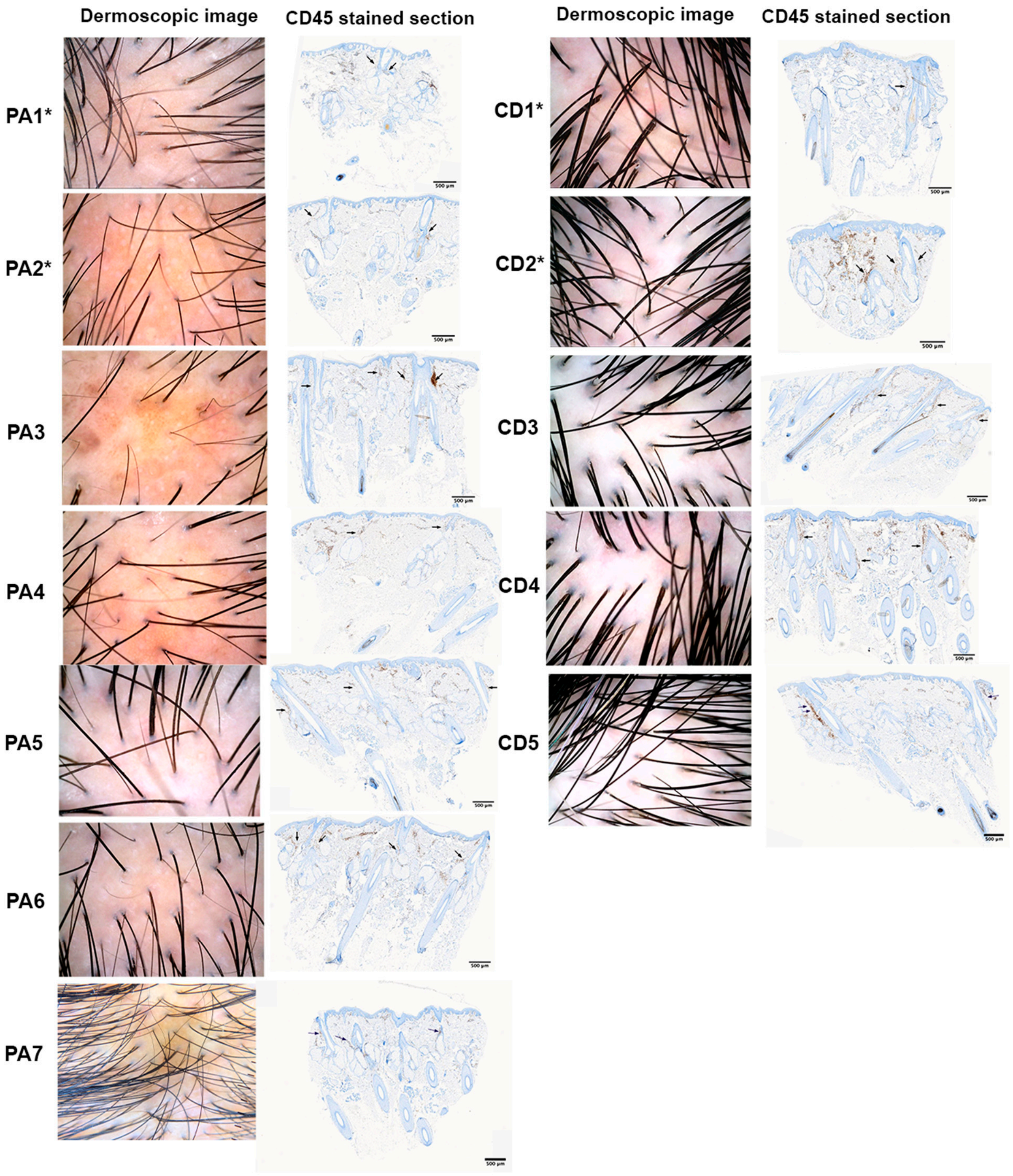

Supplementary Figure S1. Dermoscopic findings of the biopsied areas with corresponding CD45-stained sections of the PAs and CDs. Asterisks indicate patients whose tissues were subjected to spatial transcriptome profiling. Black arrows indicate CD45+ cells in PII region. PA, patient with AGA; CD, control donor.

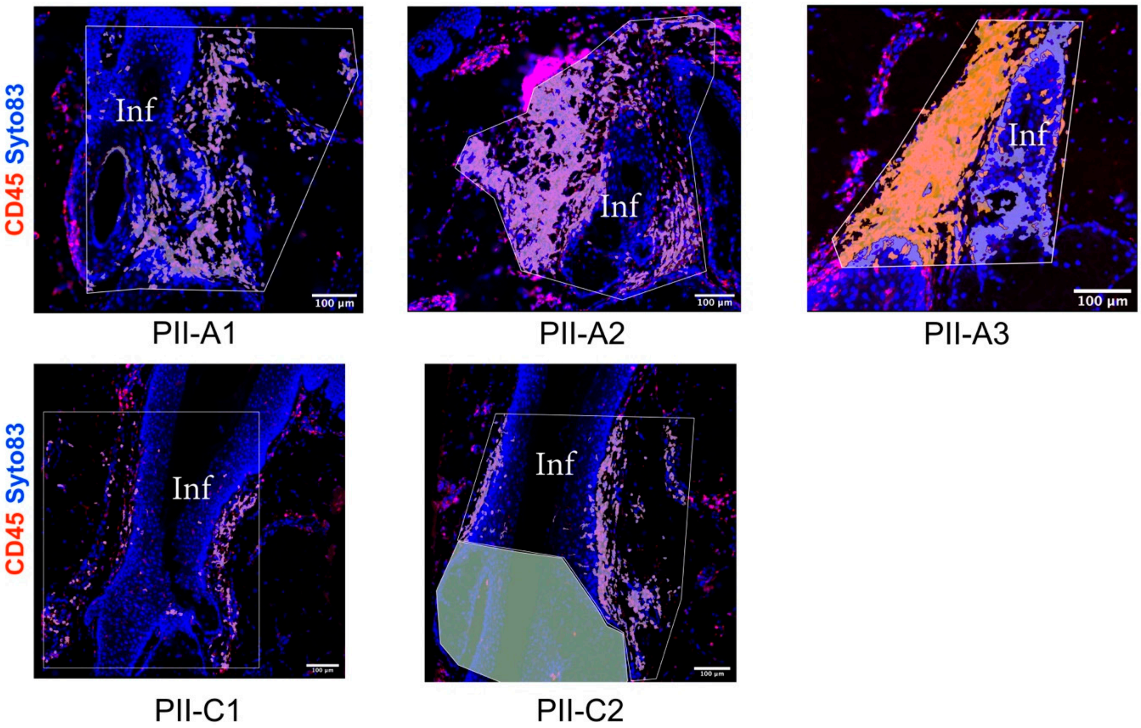

Supplementary Figure S2. Images of ROI selection for PII-A and PII-C at the lower portion of infundibulum. Arrowheads indicate segmented areas for spatial transcriptome profiling based on CD45 detection. Scale bars are 100 µm. Inf, infundibulum

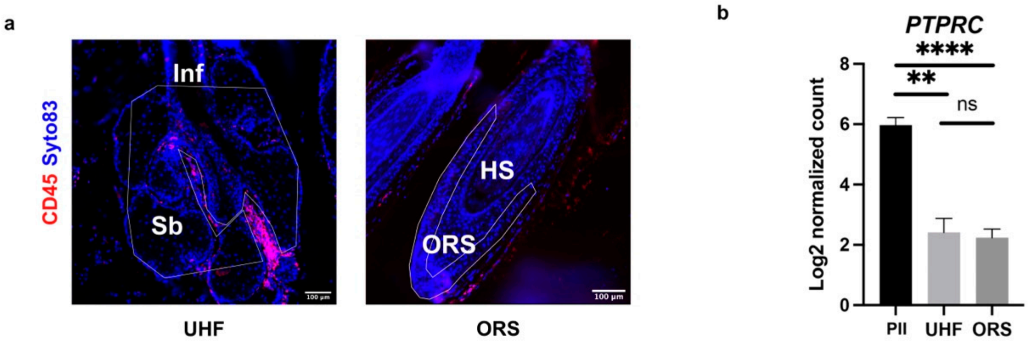

Supplementary Figure S3. Determination of the purity of ROIs. **(a)** Representative images of other ROIs, including UHF and ORS, selected for comparison. **(b)** Bar graph showing mean Log2 normalized count of *PTPRC* (CD45 encoding gene) from different types of ROIs (PII, UHF, and ORS). Level of significance: ns = not significant, **$p < 0.01$, and ****$p < 0.0001$. ORS, hair follicle outer root sheath; UHF, upper hair follicle

1.2 Supplementary Tables.

Supplementary Table S1. Patient demographic data.

ID	Age	Hamilton-Norwood classification	Underlying disease	History of noticeable scalp dermatitis	History of other hair loss disorders	History of using treatments for AGA
**CD1***	30	I (Normal)	none	none	none	none
**CD2***	28	I (Normal)	none	none	none	none
**CD3**	32	I (Normal)	none	none	none	none
**CD4**	28	I (Normal)	none	none	none	none
**CD5**	37	I (Normal)	none	none	none	none
**PA1***	27	III vertex	none	none	none	none
**PA2***	27	III vertex	none	none	none	none
**PA3**	31	III vertex	none	none	none	none
**PA4**	29	III vertex	none	none	none	none
**PA5**	30	III vertex	none	none	none	none
**PA6**	32	III vertex	none	none	none	none
**PA7**	41	III vertex	none	none	none	none

* Patients in this study. Asterisks indicate patients whose tissues were subjected to spatial transcriptome profiling. The tissues of all the patients were used for immunohistochemistry analysis. PA, patient with AGA; CD, control donor.

Supplementary Table S2. GSEA of the ranked list of genes from the expression dataset of PII-A compared with PII-C based on GOBP gene sets with FDR < 0.1.

Name	size	NES	FDR
GOBP_CYTOPLASMIC_TRANSLATION	140	2.0192	0
GOBP_REGULATION_OF_INTRINSIC_APOPTOTIC_SIGNALING_PATHWAY_BY_P53_CLASS_MEDIATOR	25	1.8838	0.0284
GOBP_REGULATION_OF_T_CELL_MEDIATED_IMMUNITY	69	1.8534	0.0412
GOBP_T_HELPER_17_CELL_DIFFERENTIATION	19	1.7707	0.0764
GOBP_CD4_POSITIVE_ALPHA_BETA_T_CELL_DIFFERENTIATION	66	1.7991	0.0788

GOBP_COTRANSLATIONAL_PROTEIN_TARGETING_TO_MEMBRANE	18	1.8064	0.0789
GOBP_INTRINSIC_APOPTOTIC_SIGNALING_PATHWAY_BY_P53_CLASS_MEDIATOR	64	1.8154	0.0811
GOBP_POSITIVE_REGULATION_OF_T_CELL_MEDIATED_IMMUNITY	47	1.7571	0.0817
GOBP_REGULATION_OF_ADAPTIVE_IMMUNE_RESPONSE	146	1.7715	0.0827
GOBP_RIBOSOMAL_LARGE_SUBUNIT_BIOGENESIS	66	1.7601	0.0850
GOBP_POSITIVE_REGULATION_OF_T_CELL_MEDIATED_CYTOTOXICITY	23	1.7442	0.0869
GOBP_REGULATION_OF_T_CELL_MEDIATED_CYTOTOXICITY	30	1.7779	0.0918
GOBP_REGULATION_OF_CD4_POSITIVE_ALPHA_BETA_T_CELL_ACTIVATION	50	1.7715	0.0919
GOBP_MATURATION_OF_LSU_RRNA	25	1.7446	0.0923
GOBP_POSITIVE_REGULATION_OF_ADAPTIVE_IMMUNE_RESPONSE	90	1.7481	0.0925
GOBP_REGULATION_OF_CD4_POSITIVE_ALPHA_BETA_T_CELL_DIFFERENTIATION	38	1.7798	0.0993

NES, normalized enrichment score.

Supplementary Table S3. Estimated cell proportions obtained by SpatialDecon deconvolution algorithm.

Cell type	PFI-C1	PFI-C2	PFI-A1	PFI-A2	PFI-A3
macrophages	0.14496991	0.13779235	0.1281776	0.05494157	0.21127193
mast	0.00421579	0.00525161	0.00825835	0.00275995	0.00282983
B.naive	0	0.02029328	0.02049359	0	0
B.memory	0	0	0	0.01989045	0
plasma	0.0328131	0.01342518	0	0.00053195	0
T.CD4.naive	0	0	0	0	0
T.CD4.memory	0.09987966	0.1002497	0.36180184	0.41895986	0.23732409
T.CD8.naive	0	0	0	0.00888485	0
T.CD8.memory	0.5049099	0.46931351	0.04709141	0.0371929	0.17221453
NK	0.04092069	0.03321029	0.0219934	0.00918498	0.01861226
pDCs	0.00825961	0.00647758	0.00270334	0.00597815	0.01210288
mDCs	0.02465289	0.03233412	0.0991721	0.03109851	0.06533577
monocytes.C	0	0	0	0	0
monocytes.NC.I	0.00565383	0.02757446	0	0	0.01460792
neutrophils	0.01568227	0.01415039	0.0272513	0.01934628	0.01005763
Treg	0.03910547	0	0.11602796	0.32663081	0.17092059
endothelial.cells	0.02877772	0.0450842	0.02791044	0.01308512	0.00714675
fibroblasts	0.05015915	0.09484334	0.13911867	0.05151464	0.07757581

Supplementary Table S4. Differential expression of different types of cells in PII-A and PII-C.

Cell type	Mean of PFI-A	Mean of PFI-C	P value
macrophages	0.1315	0.1414	0.846796
mast	0.004616	0.004734	0.955427
B.naive	0.006831	0.01015	0.812755
B.memory	0.00663	0	0.42265
plasma	0.000177	0.02312	0.254419
T.CD4.naive	0	0	
T.CD4.memory	0.3394	0.1001	0.046718
T.CD8.naive	0.002962	0	0.42265
T.CD8.memory	0.0855	0.4871	0.005817
NK	0.0166	0.03707	0.040327
pDCs	0.006928	0.007369	0.890986
mDCs	0.0652	0.02849	0.199334
monocytes.C	0	0	
monocytes.NC.I	0.004869	0.01661	0.46564
neutrophils	0.01889	0.01492	0.509362
Treg	0.2045	0.01955	0.089187
endothelial.cells	0.01605	0.03693	0.170293
fibroblasts	0.0894	0.0725	0.656808

Supplementary Table S5. DEGs with corresponding log2FC and p-value.

Gene symbol	log2FC	p-value	Regulated
HAL	2.40791812	0.04463091	Up-Regulated
MRPL40	2.36093397	0.00848932	Up-Regulated
MFHAS1	2.20543042	0.02501311	Up-Regulated
BCL11A	2.08114207	0.03292678	Up-Regulated
LIPA	1.97445752	0.02803018	Up-Regulated
EVC2	1.97179333	0.03322541	Up-Regulated
POLR3E	1.89437079	0.02770044	Up-Regulated
RGMB	1.7794576	0.03641506	Up-Regulated
EGLN3	1.77732357	0.0228889	Up-Regulated
C1orf100	1.74544906	0.01216533	Up-Regulated
TMSB4X	1.62713295	0.02583509	Up-Regulated
SH3RF1	1.59964729	0.00732259	Up-Regulated
REXO2	1.59627153	0.04255146	Up-Regulated
NANS	1.59148458	0.04342674	Up-Regulated
SLC6A9	1.55506349	0.01624198	Up-Regulated
SPIB	1.55377289	0.03406352	Up-Regulated
USP13	1.54527241	0.00798996	Up-Regulated
CBFB	1.51094508	0.03335368	Up-Regulated

GRIN2A	1.5091314	0.02077795	Up-Regulated
HLA-DRA	1.50654151	0.04353169	Up-Regulated
CSMD3	1.50548157	0.04930965	Up-Regulated
SCN2B	1.50439579	0.01916509	Up-Regulated
ZNF280B	1.48962229	0.04771711	Up-Regulated
CCNI2	1.48492357	0.00463944	Up-Regulated
NUP35	1.44762056	0.01384314	Up-Regulated
MMP28	1.4409255	0.01906216	Up-Regulated
ADAM15	1.42596168	0.00074743	Up-Regulated
ENPP2	1.42510984	0.00303093	Up-Regulated
CD40	1.41891392	0.02886742	Up-Regulated
IL1RAP	1.40429555	0.04328716	Up-Regulated
TGFB3	1.4041654	0.01781829	Up-Regulated
IL6R	1.39591522	0.02706017	Up-Regulated
CHCHD7	1.38642592	0.01359527	Up-Regulated
FAM216A	1.35846104	0.04790002	Up-Regulated
GNG13	1.32295562	0.02435551	Up-Regulated
SYNGR2	1.31856242	0.03268772	Up-Regulated
MAN1C1	1.31735669	0.02641336	Up-Regulated
STING1	1.30135762	0.02825094	Up-Regulated

UQCC2	1.29660569	0.03844327	Up-Regulated
ZNF154	1.29191915	0.01144712	Up-Regulated
DNPH1	1.27811085	0.01632337	Up-Regulated
PRKAR2B	1.27239786	0.03019356	Up-Regulated
SSX2IP	1.25538937	0.00736139	Up-Regulated
PRDX3	1.24955951	0.02672437	Up-Regulated
FGF6	1.24023538	0.01826223	Up-Regulated
CMTM7	1.23150554	0.01048358	Up-Regulated
NAA60	1.22247489	0.01335074	Up-Regulated
ARL14EP	1.21958807	0.0053003	Up-Regulated
SDCCAG8	1.21712823	0.02843853	Up-Regulated
PTPN5	1.21035651	0.04840172	Up-Regulated
MZT2B	1.20058356	0.00459528	Up-Regulated
IL1R2	1.19386049	0.04140933	Up-Regulated
ROBO1	1.18948224	0.03591052	Up-Regulated
ECD	1.18111858	0.03024754	Up-Regulated
FAM177A1	1.16897358	0.03490541	Up-Regulated
CD4	1.16845795	0.01516977	Up-Regulated
HEXD	1.1529132	0.00680421	Up-Regulated
KLHDC7B	1.15255724	0.02840892	Up-Regulated

TIFA	1.15255724	0.02840892	Up-Regulated
TSKU	1.1511189	0.04649426	Up-Regulated
FN3KRP	1.14795531	0.02067302	Up-Regulated
ZMAT4	1.14795531	0.03005874	Up-Regulated
RNF103	1.14010695	0.00744189	Up-Regulated
MEA1	1.1392735	0.02908376	Up-Regulated
HAPLN3	1.13901282	0.04575384	Up-Regulated
PPIA	1.13596554	0.01496461	Up-Regulated
UBE2Q1	1.13588054	0.0405314	Up-Regulated
HEPACAM	1.13578002	0.01756006	Up-Regulated
NUBPL	1.13129615	0.01174727	Up-Regulated
MAT2A	1.13030698	0.04019443	Up-Regulated
XYLT1	1.11442079	0.0091201	Up-Regulated
NARS1	1.11180597	0.0388523	Up-Regulated
SFRP5	1.10611168	0.03971525	Up-Regulated
RUBCN	1.1007733	0.02466354	Up-Regulated
TAPBP	1.09736699	0.00643208	Up-Regulated
NCOA7	1.09481193	0.02444234	Up-Regulated
SDK2	1.09469867	0.00588998	Up-Regulated
HARS1	1.09469867	0.03493585	Up-Regulated

RAB7A	1.08581636	0.01488937	Up-Regulated
GSPT1	1.08180906	0.03486715	Up-Regulated
HMOX2	1.08151692	0.0201503	Up-Regulated
HMGXB3	1.07878239	0.0105596	Up-Regulated
TP53BP2	1.07440216	0.00208379	Up-Regulated
GGH	1.07332189	0.02465152	Up-Regulated
RAB38	1.07147983	0.03581979	Up-Regulated
FAM207A	1.07058173	0.02591574	Up-Regulated
PRKAG1	1.07028916	0.02950415	Up-Regulated
RAPGEF5	1.06664919	0.02616191	Up-Regulated
SCGB2B2	1.05871746	0.04017688	Up-Regulated
DCDC1	1.05863711	0.02806857	Up-Regulated
C8orf88	1.05508803	0.01250126	Up-Regulated
ANKRD52	1.05192137	0.03387652	Up-Regulated
LARP4B	1.03529928	0.02398499	Up-Regulated
ANXA11	1.02999212	0.01617136	Up-Regulated
NOL7	1.02972964	0.006066	Up-Regulated
SHMT2	1.02921955	0.03514398	Up-Regulated
SNRPE	1.02838589	0.02502842	Up-Regulated
CEP68	1.02242964	0.02639157	Up-Regulated

MGAT5	1.01874282	0.02889633	Up-Regulated
FUT3	1.0132444	0.02867008	Up-Regulated
ATE1	1.0128026	0.04292952	Up-Regulated
CBX6	1.00797071	0.02512632	Up-Regulated
PACSIN1	1.00522924	0.0393601	Up-Regulated
CANX	1.00250533	0.00789074	Up-Regulated
SPATA3	1.00215354	0.03216551	Up-Regulated
SNX20	1.00122992	0.00836741	Up-Regulated
MAN1A1	-1.0022283	0.04622801	Down-Regulated
FAT1	-1.0106491	0.00755908	Down-Regulated
TMEM91	-1.0154133	0.02196458	Down-Regulated
BSN	-1.0178013	0.01634593	Down-Regulated
BMERB1	-1.028212	0.02145741	Down-Regulated
TRIM22	-1.039083	0.0236289	Down-Regulated
LRRC42	-1.0438808	0.02522087	Down-Regulated
SLC30A5	-1.0673184	0.04717404	Down-Regulated
FSTL3	-1.0680355	0.01321471	Down-Regulated
LY6E	-1.0722776	0.01952366	Down-Regulated
FDPS	-1.0851081	0.0133718	Down-Regulated
ITGB6	-1.0855246	0.00857119	Down-Regulated

ATP23	-1.0906291	0.00237649	Down-Regulated
ELF5	-1.0913739	0.00714369	Down-Regulated
LLGL2	-1.0963318	0.00513128	Down-Regulated
CYP2U1	-1.0976999	0.03590349	Down-Regulated
AQP1	-1.1034659	0.02353076	Down-Regulated
GPR119	-1.1095679	0.04378477	Down-Regulated
IRGC	-1.1179584	0.03455621	Down-Regulated
SUPT7L	-1.1193562	0.03629856	Down-Regulated
P2RY4	-1.1206741	0.00503428	Down-Regulated
ANKRD20A1	-1.1266597	0.02252638	Down-Regulated
CXCR3	-1.1453182	0.04449163	Down-Regulated
SPAST	-1.1487687	0.04370214	Down-Regulated
APMAP	-1.1549233	0.00570208	Down-Regulated
ANKRD22	-1.1645131	0.01747974	Down-Regulated
CATSPER1	-1.1685466	0.01505678	Down-Regulated
C1QB	-1.1692506	0.04199542	Down-Regulated
LARGE2	-1.1890021	0.00478819	Down-Regulated
SLIT2	-1.1966206	0.03679875	Down-Regulated
RPS6KL1	-1.207236	0.02115649	Down-Regulated
CTSG	-1.2185566	0.00542684	Down-Regulated

PLEKHB1	-1.2242193	0.04878947	Down-Regulated
HDDC2	-1.230161	0.03567155	Down-Regulated
CHST14	-1.2326376	0.00209908	Down-Regulated
MTURN	-1.2406973	0.04731319	Down-Regulated
SPAG16	-1.2513464	0.03146996	Down-Regulated
FOLR2	-1.2545509	0.01018985	Down-Regulated
OR5AK2	-1.2725672	0.01436951	Down-Regulated
ATP5MC2	-1.2908518	0.00566492	Down-Regulated
C1QC	-1.3231562	0.01063085	Down-Regulated
IL5RA	-1.3347107	0.03298094	Down-Regulated
CCN1	-1.3545427	0.01345729	Down-Regulated
OR2A1	-1.3580962	0.00998174	Down-Regulated
FADS1	-1.3606646	0.02214613	Down-Regulated
CCL4L2	-1.3730312	0.01962131	Down-Regulated
CCR5	-1.3824486	0.01441209	Down-Regulated
FADS6	-1.4188579	0.04066781	Down-Regulated
KCNK4	-1.4446167	0.03647813	Down-Regulated
KLRG1	-1.4609185	0.00457199	Down-Regulated
INSIG1	-1.4972293	0.02922464	Down-Regulated
THRSP	-1.5191894	0.02579746	Down-Regulated

ANGPTL5	-1.5261673	0.04460878	Down-Regulated
DERL3	-1.5569128	0.0066261	Down-Regulated
STAB1	-1.5849348	0.03315827	Down-Regulated
SLC40A1	-1.6425817	0.04256775	Down-Regulated
MAP7D2	-1.7051094	0.04418645	Down-Regulated
ACSL1	-1.7125422	0.03377552	Down-Regulated
PNO1	-1.7278379	0.02104743	Down-Regulated
IFI44L	-1.7436244	0.0052113	Down-Regulated
FASN	-1.7770066	0.02315505	Down-Regulated
PRF1	-1.7879778	0.01077843	Down-Regulated
PPEF1	-1.8186485	0.04570394	Down-Regulated
ACSBG1	-1.8222556	0.03444138	Down-Regulated
GZMH	-1.831351	0.00562199	Down-Regulated
GCHFR	-1.880447	0.04676455	Down-Regulated
IFI27	-1.9341095	0.01160937	Down-Regulated
CD8A	-1.9934143	0.03345857	Down-Regulated
RNASE1	-2.2474761	0.02278295	Down-Regulated
NKG7	-2.4194552	0.03278479	Down-Regulated
CYP4F8	-2.563593	0.00730499	Down-Regulated
C4B	-2.8772662	0.01290581	Down-Regulated

AGPAT4	-2.9203153	0.02501436	Down-Regulated
SCN9A	-3.1232294	0.02885467	Down-Regulated

Supplementary Table S6. Over-represented gene ontology (GO) terms

source	term_name	Adjusted p-value	Negative log10 of adjusted p-value	Term size	Query size	Intersection size
GOBP	response to chemical	0.00148451	2.82841722	4316	163	61
GOBP	response to organic substance	0.00183307	2.7368206	3009	163	47
GOBP	response to stimulus	0.00211223	2.67525955	8815	163	98
GOBP	immune system process	0.00296352	2.52819214	2748	163	43
GOBP	defense response	0.00333345	2.47710548	1687	163	31
GOBP	cellular response to cytokine stimulus	0.00355732	2.44887735	828	163	20
GOBP	response to external stimulus	0.00378231	2.42224263	2752	163	42
GOBP	response to cytokine	0.00378231	2.42224263	922	163	21
GOBP	inflammatory response	0.007966	2.09875988	756	163	18
GOBP	response to stress	0.007966	2.09875988	3780	163	51

GOBP	regulation of localization	0.00877356	2.05682424	2701	163	40
GOBP	cytokine-mediated signaling pathway	0.00877356	2.05682424	493	163	14
GOBP	cellular response to chemical stimulus	0.00878802	2.05610878	3008	163	43
GOMF	immune receptor activity	0.00658481	2.18145649	141	173	8
GOMF	cytokine binding	0.00658481	2.18145649	141	173	8
GOMF	cytokine receptor activity	0.00658481	2.18145649	99	173	7
GOCC	melanosome	0.00597953	2.22333304	109	173	7
GOCC	pigment granule	0.00597953	2.22333304	109	173	7
GOCC	cytoplasm	0.00597953	2.22333304	12193	173	123
GOCC	intrinsic component of membrane	0.00621765	2.20637378	5897	173	71

Supplementary Table S7. Top ten hub genes based on MCC scores with corresponding differential expression values.

**Gene symbol**	**Name**	MCC score	**LogFC**	**p-value**	**Regulation**
CD8A	CD8a molecule	420	-1.9934143	0.03345857	Down

CD4	CD4 molecule	415	1.16845795	0.01516977	Up
PRF1	perforin 1	385	-1.7879778	0.01077843	Down
CD40	CD40 molecule	248	1.41891392	0.02886742	Up
CCR5	C-C motif chemokine receptor 5	247	-1.3824486	0.01441209	Down
CCL4L2	C-C motif chemokine ligand 4 like 2	240	-1.3730312	0.01962131	Down
CXCR3	C-X-C motif chemokine receptor 3	192	-1.1453182	0.04449163	Down
NKG7	natural killer cell granule protein 7	120	-2.4194552	0.03278479	Down
HLA-DRA	major histocompatibility complex, class II, DR alpha	61	1.50654151	0.04353169	Up
C1QC	complement C1q C chain	50	-1.3231562	0.01063085	Down

Supplementary Table S8. Predicted upstream regulators with p-value of overlap of less than 0.05 and absolute z-score ≥ 2

Upstream Regulator	Molecule Type	Predicted Activation State	Activation z-score	p-value of overlap	Target Moleules in Dataset
LEF1	TF	Activated	2	0.0084	MGAT5,NCOA7,PRF1,SH3RF1
IL13	CK	Activated	2.121	0.0035	ADAM15,CD40,CHCHD7,ENPP2,FADS1, IL1R2,LIPA,SYNGR2
TBX21	TF	Inhibited	-2.204	0.0000	CCR5,CXCR3,IL6R,KLRG1,NKG7,PRF1

NONO	TF	Inhibited	-2.219	0.0039	CCL4L1/CCL4L2,IFI27,IFI44L,LY6E,TRIM22

Supplementary Table S9. Antibodies used for immunohistochemistry. Staining protocols automated = BenchMark ULTRA IHC/ISH System - Roche Diagnostics.

Primary antibody	Catalog	Manufacturer	Concentration	Staining protocol	Primary antibody titer
CD45	760-2505	Ventana, Roche	1 ug/ml	Automated	1:50
CD4	104R-16	Sigma-Aldrich	24.2 ug/ml	Automated	1:25
CD8	108M-96	Sigma-Aldrich	43 ug/ml	Automated	1:50
FOXP3	14-4777-82	ThermoFisher	0.5 mg/ml	Automated	1:50
IFN-γ	ab267369	Abcam	10 ug/ml	Automated	1:50
IL13	orb10895	Biorbyt	5 ug/ml	Automated	1:100
T-bet	14-5825-82	ThermoFisher	5 ug/ml	Automated	1:100
GATA3	MA1-028	ThermoFisher	1 ug/ml	Automated	1:1000
CD56	156R-9	Sigma-Aldrich	concentrated	Automated	1:100

IL-17A	PA5-79470	ThermoFisher	0.5 mg/ml	Automated	1:1000
